# Developing a Center for Education Research and Scholarship in a Department of Pediatrics

**DOI:** 10.15694/mep.2020.000181.1

**Published:** 2020-08-27

**Authors:** Daniel Nicklas, Meghan Treitz, Stephen Daniels, Janice Hanson

**Affiliations:** 1 University of Colorado School of Medicine; 2 Washington University in St. Louis

**Keywords:** Medical education, education research, education scholarship, faculty development

## Abstract

This article was migrated. The article was marked as recommended.

Background

The skills needed to engage in scholarship in medical education are not part of the training that all physicians acquire. To build skills and promote scholarship, we developed a Center for Education Research and Scholarship (CERS) in the Department of Pediatrics at the University of Colorado. In this paper, we offer recommendations for others who seek to establish departmental-level efforts to support education.

Approach and Lessons Learned

CERS provides an “education home” for those interested in education scholarship, supplementing campus-wide efforts such as an Academy of Medical Educators. Mentorship from two experienced leaders in medical education provided a foundation for other faculty in the department and helped to build scholarship efforts more broadly. Through a weekly meeting and an annual departmental retreat, CERS provides opportunities for community among educators, faculty development in the skills needed to engage in education research, and a forum for generating ideas and planning projects.

Essential resources for success include at least one leader with expertise in educational research, an administrative and/or research assistant, and some funding for faculty time and initiation of projects. Mentors with experience in education research and scholarship are also needed, although a group of mentors quickly grows as more individuals engage.

Results

Benefits to the department include peer-reviewed presentations and publications in medical education, with regional, national, and international recognition. Faculty members can focus on medical education as a key component of their careers, and the quality of education programs is enhanced.

Conclusions

While it takes time to fully develop a departmental center for education scholarship, it is possible to start small and grow. One or two leaders in education with vision can begin the effort and engage others, and the faculty will begin to experience the satisfaction of collaborative projects in education, successful innovation, and dissemination of scholarship.

## Background

As knowledge about human health and disease has expanded, technology has developed exponentially, and health care systems and financing have become more complex, the apprenticeship model of medical education, in which physicians teach medicine to apprentices who work closely with them for years, has faded away. At the same time, the science of education has also grown considerably, with conceptual frameworks ( Klein and Gusic, 2019; Samuel et al., 2019; Varpio et al., 2019), pedagogy, and strategies that are very relevant to medical education ( Li et al., 2019). From these changing contexts, medical educators have realized that it is important to study what we do rigorously and learn what works best for education in today’s healthcare world ( Sullivan, 2011).

The skills needed to systematically approach scholarship in medical education are not part of the training that all physicians acquire to practice medicine. And it’s hard for individual physicians who have access to few resources to do research and scholarship in education. Teachers and scholars need new skills and the support of resources and colleagues to do the education research needed to gather evidence about teaching and learning among physicians. We need to know, for example, 1) What approaches to education result in effective learning (the scholarship of discovery about teaching and learning)? 2) How effective are the ongoing teaching in medical education, the ways we assess the progress of medical learners, and the new curricula that we create (the scholarship of teaching)? 3) How can we effectively apply new approaches to education to the challenges of new knowledge and new needs in clinical settings (the scholarship of application)? 4) What are useful ways to integrating science from different fields that is relevant to education (the scholarship of integration) ( Glassick, 2000)?

Faculty members at academic medical centers, however, face many challenges that make it difficult to pursue education research and scholarship. The culture at academic medical centers tends to support and reward basic science and clinical research more so than education research ( Irby and O’Sullivan, 2018). Some of the needs faced by faculty doing education scholarship include finding local networks of support for this work ( Peterson et al., 2019), learning to apply education theory to curriculum development and other scholarship projects ( Johnston, Bennett and Kajamaa 2018; Laksov, Doman and Teunissen, 2017), navigating Institutional Review Boards ( DeMeo, Nagler and Heflin, 2016), and using education research literature in meaningful ways ( Wilson and Brame, 2018). Many medical schools have developed campus-wide offices to support medical education research and scholarship and address these needs ( Humphrey-Murto et al., 2020; Thammasitboon et al., 2017; Cofrancesco et al., 2018). There is also a need for support for education research and scholarship at the department level, particularly in large departments with many faculty members who are engaged in the work of teaching, but are siloed from other resources on campus. There are fewer examples of departmental centers or offices for medical education research and scholarship. We found three published examples of departmental-level units to support education scholarship, one each in internal medicine ( Beckman, Lee and Ficalora, 2009), anesthesia/critical care ( Schwengel and Toy, 2019), and pediatrics ( Klein et al., 2020). The purpose of this paper is to describe the experience of building a Center for Education Research and Scholarship (CERS) in the Department of Pediatrics at the University of Colorado, and to offer recommendations for others who seek to establish departmental-level efforts to support education scholarship in academic medical centers.

## Challenges in Education Research and Scholarship

Traditionally, physicians have taught spontaneously in clinical settings, created lectures, developed teaching materials, and implemented curricula with little or no specific training in medical education. Medical education innovations were published with minimal data and weak outcomes, with reported outcomes often involving learner acceptance of the activity (“I liked it”) and self-reported improvements in confidence or knowledge. Today, manuscript acceptance to medical education journals depends on evidence of rigorous scholarship, organization around conceptual frameworks, and stronger outcomes, such as demonstrated changes in the behavior of learners.

While some faculty pursuing medical education as academic scholarship have additional training in medical education through certificate or degree programs, such as the Education Scholars Program ( Jerardi et al., 2016; Baldwin, Gusic and Chandran, 2017), most departments have many faculty members without expertise in these skills, but who participate at varying levels in creating, implementing, and/or evaluating education programs. Faculty need professional development learning opportunities and mentorship for planning, leading, and participating in education research.

Outside sources of funding for medical education research are limited, and most do not include faculty salary support. This leaves education researchers dependent on section or division support for time, which is rarely provided, or requires researchers to complete education research during personal time. This situation is not conducive to improving existing educational programs or innovating within educational, which is regrettable both for programs in institutions and for the greater medical education community.

Most academic institutions have clinical and translational research centers, which provide research support, including administrative support with protocol development, budgeting assistance, assistance with study implementation, as well as clinical research services. Education scholarship is different than traditional “research” and it is difficult to simply “piggy-back” onto the existing research administrative pathways. Little to no administrative support exists for faculty applying for Institutional Review Board (IRB) approval and grants in education. These administrative pathways are complicated and time consuming, which is compounded even further for faculty without protected time (or salary support) for this research. It can also be difficult for individual researchers to interface with the institutional grant office, which usually has little understanding of education grants. One major factor encountered is that education grants tend not to allow indirect costs. This is viewed negatively, as these costs represent additional expenses of using the institution’s facilities and support for the project. Our institution has also reported challenges and confusion with flow of grant money to the grant office and subsequently to the project. These administrative complexities necessitate support staff who are intimately familiar with the nature of education projects and the workings of the associated IRB and grant processes-and who have the time needed to work through them.

Even within one academic department, such as pediatrics, it can be common for people to work in silos. Office spaces are separate, schedules are complex, and there is little time for networking. CERS provides an “education home” for those interested in education scholarship. At the University of Colorado, CERS originated with leadership from our Vice Chair of Education and the Director for Education Research and Scholarship in the Department of Pediatrics. The Vice Chair brought expertise in patient care and medical education, while the director of the center brought PhD expertise in education research. Their experience producing and disseminating medical education scholarship locally, nationally, and internationally has provided a foundation for mentorship of other faculty in the department, which facilitates scholarship more broadly.

## A Departmental Center for Education Research and Scholarship (CERS)

CERS is structured to build community. A pre-existing weekly meeting, called the Pediatric Education Group (PEG), has become the environment for CERS educators to share works in progress, train colleagues on particular areas of expertise (e.g., building validity evidence), and provide an open forum for generating ideas and planning projects. In addition, it has become a meeting for planning an annual departmental education retreat that focuses on “hot topics” in medical education and showcasing educational research and scholarship from the department. These retreats are open to educators from all schools and departments on campus. The retreats provide an opportunity for all of our educators to learn about the skills, expertise, and possibilities for collaboration here in our own department.

In addition, CERS legitimizes a structure within the Department of Pediatrics for people to work collaboratively. Our website (Department of Pediatrics, 2019) creates a home base for scholars to connect to resources they need, including the CERS team, mentors for their projects, a schedule of events including the PEG meeting, a list of publications in medical education scholarship from our own faculty and residents, and a list of funding opportunities (internal and external). The design of the website includes a professionally designed compass symbol for easily recognizable branding that promotes our tenets of education, research, evaluation, and scholarship.

Lastly, while there exists an Academy of Medical Educators ( Corral, Guiton and Aagaard, 2017) on the campus, CERS serves to solidify the education scholarship within the Department of Pediatrics, complement the resources of the Academy, and provide a bridge to the wider campus. CERS provides a forum within which there is a familiarity with the work and culture of the department. This serves to make connections and increase collaboration where it is close in time and place to the work people do. This departmental hub also complements faculty development that is done through the Academy by focusing on the needs at the local level within our department. The CERS leadership are members of the Academy and serve as a bridge by advertising medical education grand rounds, making connections with others on campus, and asking people with expertise in other departments to come teach the faculty in pediatrics about their work and skill sets.

## Lessons Learned

In developing CERS at the University of Colorado, we have identified essential resources for success. The Center needs to have at least one leader with expertise in educational research, and this person needs an administrative assistant. Mentors with experience in education research and scholarship are needed, although one or two mentors can support an initial group of faculty scholars, and the group of mentors quickly grows as more individuals gain experience. Additional faculty with some salary support help maintain the activities of the Center, including review of grant proposals and pairing new educational researchers with appropriate project mentors. As projects develop, the Center can build and maintain a community of scholars. There are many ways to accomplish this, but we have focused on a weekly meeting for educators to share their work and learn about education topics as a group (PEG). The yearly medical education retreat also builds the community of scholars, focusing on one theme in medical education each year with keynote speakers and breakout workshops.

A full time research assistant who helps with preparing IRB and grant proposals, as well as assisting with various aspects of research projects, increases the pace of project development tremendously. Departmental funding also allows us to set up a mini-grant program to provide small monies to support projects, which provides another boost to faculty who start new projects.

Technology is important to the Center also. We have benefited from appropriate systems (including a Learning Management System), software (such as EndNote and software for qualitative and quantitative data analysis), and a website. A functional website allows for people to connect with the center and learn about current areas of focus. An education scholarship repository is helpful for tracking projects and helping educators with similar areas of interest connect and collaborate. A database for connecting people is also useful. Additional staff support helps with more extensive and sophisticated project development, for example in instructional design, educational technology, and general technology support. Finally, as with all new enterprises, careful branding and promotion helps to secure the Center as a recognized constituent part of the department.

Benefits to the department include a long list of peer-reviewed presentations and publications in medical education, with regional, national, and international recognition for the work we have done in education. (See the Table 1 below) Faculty members have been able to focus on medical education as a key component of their careers, and many have been promoted in large part based on their work in education. The quality of our education programs has been enhanced, too-with the evidence that a scholarly approach produces, and the scholarship that enables us to share innovations with others in the medical education community beyond our institution.

**Table 1.** Selected projects through the Center for Education Research and Scholarship

Projects include curriculum development, education research, and novel approaches to teaching and evaluation.	Select scholarly products include peer-reviewed abstracts, workshops, symposium, and publications
Effects of frequently-changing attendings on medical students’ learning	**Seltz LB, Montgomery A, Lane JL, Soep J, Hanson JL.** Medical students’ experiences working with frequently rotating pediatric inpatient attending physicians. Hosp Pediatr.
Interns’ learning during rounds	**Seltz LB, Preloger E, Hanson JL, Lane L.** Ward Rounds With or Without and Attending Physician: How Interns Learn Most Successfully. Academic Pediatr.
The development of an academic half-day	**Zastoupil L, McIntosh A, Sopfe J, Burrows J, Kraynik J, Lane L, Hanson J, Seltz LB.** Positive Impact of Transition from Noon Conference to Academic Half Day in a Pediatric Residency Program. Acad Pediatr.
Stereotype threat in core clerkships	Bullock J, **Lockspeiser T**, del Pino-Jones A, Richards R, Teherani A, Hauer K. They don’t see a lot of people my color: a mixed-methods study of racial/ethnic stereotype threat among medical students on core clerkships. Acad Medicine.
Education in Pediatrics Across the Continuum	Andrews JS, Bale JF Jr, **Soep JB**, Long M, Carraccio C, Englander R, Powell D; EPAC Study Group. Education in Pediatrics Across the Continuum (EPAC): First Steps to Realizing the Dream of Competency-Based Education. Acad Med.
Culture of reflection	**Treitz M, Hanson J.**Promoting a culture of Reflection in a Department of Pediatrics. Oral Presentation at AMEE International Association for Medical Education Meeting.
High Value Care curriculum for sub-interns	**O’Hara K, Burch A, Baca M, Ziniel S, Soep J, Treitz M.**A High Value, Cost-Conscious Medicine Curriculum for Pediatric Sub-Interns. Poster presentation at Pediatric Academic Societies Meeting, San Francisco. May 2017.
Pediatric Primary Care Curriculum	**Nicklas D, Lane JL, Hanson JL.** If You Build It, Will They Come? A Hard Lesson for Enthusiastic Medical Educators Developing a New Curriculum. J Grad Med Educ.
Validity Evidence for assessments of team member performance and team functioning in an interprofessional course	Paul CR, Ryan MS, Dallaghan G, Jirasevijinda T, Quigley PD, **Hanson JL**, Khidir AM, Petershack J, Jackson J, Tewksbury L, & Rocha M. Collecting Validity Evidence: A Hands-on Workshop for Medical Education Assessment Instruments. MedEdPORTAL
Defining best practices for the consultation/referral process in pediatrics	Hamburger EK, **Lane JL**, Agrawal D, Boogaard C, **Hanson JL**, Weisz J, Ottolini M. The referral and consultation entrustable professional activity: defining the components in order to develop a curriculum for pediatric residents. Academic Pediatr. Muradian S, Widge A, **Hanson JL**, **Lane JL**, Boogaard C, Agrawal D, Ottolini M, Hamburger EK. Tools for Learning About the Referral and Consultation Process for Pediatric Residents. Academic Pediatr.
Defining best practices for the consultation/referral process in pediatrics-parents’ perspectives	**Stempel H, Hanson J**, Hamburger E, Ottolini M, **Lane JL**. “How many parents would have given up?” Caregiver insights on the emotional impact of the specialty referral process to inform resident education.” Pediatric Academic Societies Meeting.
Advocacy curriculum	**Treitz M.** An Advocacy and Reflective Practices Curriculum: Changing the Culture in a Department and Residency to Advance Professional Ideals and Practice in Educating and Training to Professionalism Monograph.
Poverty Curriculum	**Kaferly J, McClure C, Stempel H**, Chamberlain L, **Lane JL**. “We can try as much as we to be an advocate:” Pediatric resident experiences and perspectives related to delivering care to children in poverty. Pediatric Academic Societies Meeting.

## Conclusions

The Center for Education Research and Scholarship in the Department of Pediatrics at the University of Colorado developed to its current shape over eight or ten years. It is certainly possible to start small and grow, or to start with fewer resources in a smaller department. One or two leaders in education with vision can begin the effort, starting with vision for education research and scholarship, an administrative assistant, some funds for software and mini-grants for projects, and a website to let the faculty know about the new and emerging activities and opportunities in education. From our experience, we would say, it is well worth the effort!

## Take Home Messages

• Traditionally, skills needed for scholarship in medical education are not a part of medical training
• A center for education scholarship within a department offers an “education home” for those interested in education scholarship
• A center for education scholarship provides infrastructure to help procure funding, IRB approval, and appropriate mentorship
• Essential resources include at least one leader with expertise in educational research, an administrative and/or research assistant, and some funding for initiation of projects
• Benefits include peer-reviewed presentations and publications and regional, national, and international recognition for medical education work

## Notes On Contributors

Daniel Nicklas is an Associate Professor and the Director for Primary Care Education for the Pediatric Residency Program, University of Colorado School of Medicine. ORCID: https://orcid.org/0000-0003-4550-5160

Meghan Treitz is an Associate Professor of Pediatrics and the Associate Director for the Pediatric Clerkship, University of Colorado School of Medicine. ORCID: https://orcid.org/0000-0002-4648-9468

Stephen Daniels is a Professor and the Pediatrician in Chief in the Department of Pediatrics, University of Colorado School of Medicine. ORCID: https://orcid.org/0000-0001-5520-6952

Janice Hanson is a Professor of Medicine and Director of Education Scholarship Development at Washington University in St. Louis School of Medicine. ORCID: https://orcid.org/0000-0001-7051-8225
