# Supplementary material for: Developing a Center for Education Research and Scholarship in a Department of Pediatrics
Source: MedEdPublish (2016). 2020 Aug 27;9:181. [Version 1] doi: 10.15694/mep.2020.000181.1 (PMC10702635; doi:10.15694/mep.2020.000181.1)
Supplement: Peer review history

# Reviewer report: Reviewer response for version 1

**DOI:** 10.21956/mep.20110.r31009

**Published:** 2020-10-05

**Authors:** Ken Masters

**Affiliations:** 1 Sultan Qaboos University

This review has been migrated. The reviewer awarded 3 stars out of 5 An interesting paper dealing with developing a Centre for Education Research and Scholarship in a Department of Pediatrics. The authors establish the background needs stemming from the changes that have occurred in medical education of the past few decades, and the resultant need for a systematic approach to developing education research and scholarship. This is in the context of medical education’s being seen as the poor cousin to “hard science”.Although the specifics of the situation may differ from medical school to medical school, the concerns raised in the paper are recognisable by anyone working in medical education. The establishment of the centre is clearly and succinctly described, and the success of the centre is demonstrated by the presentations and publications.There is, however, the old adage that failure is an orphan and that success has many parents (or many people who claim to be parents), so the direct link between the Centre and the publications needs to be clarified. I think, for a reader to gauge the level of success, then I would like to see, for at least three of these publications, a short paragraph describing exactly what benefit was derived from the Centre, and how its existence and functions contributed to them. This would be important, not only for this paper, but, if a reader took this paper to an administrator as part of the motivation to have such a Centre, the response might be: “How do we know that these papers would not have written anyway, even if there were no such Centre?” While that question is too hypothetical to be answered with 100% certainty, it would be useful to trace the direct significant contribution of the centre to the projects.I look forward to a Version 2 of the paper in which this aspect is expanded somewhat.

Reviewer Expertise:

NA

No decision status is available

# Reviewer report: Reviewer response for version 1

**DOI:** 10.21956/mep.20110.r31007

**Published:** 2020-09-08

**Authors:** P Ravi Shankar

**Affiliations:** 1 American International Medical University

This review has been migrated. The reviewer awarded 4 stars out of 5 I enjoyed reading this article about developing a centre for education research and scholarship in the department of paediatrics at a medical school in the United States. I do agree with the authors that medical education research and scholarship has not been prioritized. Most medical school faculty still do not have formal training in medical education. In many parts of the word, the FAIMER fellowship is providing a community of practice. Obtaining protected time for research remains a challenge and funding for education research is still limited. The experiences of the authors will be of relevance and interest to educators in a diversity of settings.

Reviewer Expertise:

NA

No decision status is available

# Reviewer report: Reviewer response for version 1

**DOI:** 10.21956/mep.20110.r31008

**Published:** 2020-09-05

**Authors:** Felix Silwimba

**Affiliations:** 1 University of Lusaka

This review has been migrated. The reviewer awarded 5 stars out of 5 The manuscript is about creating medical education awareness amongst faculty It is original and currentThe abstract is a good summary of the contents.The strengths of this manuscript are that it clearly recognizes the shortfall of traditional medical education by apprenticeship, that like all established traditions is difficult to die out. The traditional methods lack scholarship in medical education. The advances in medical technologies and educational technologies requires that medical educators be qualified and be in a specialty. It also identifies the lack of research funding in preference for funding basic sciences and clinical medicine.The conclusion is concise and very encouraging to a medical education enthusiast. it encourages the reader to start small within a department, then grow to be multi-departmental, national, and international. Personally, I find this manuscript motivating, as I’m a medical educator struggling to have my voice heard in low income country. My desire is to one day establish a medical education unit.

Reviewer Expertise:

NA

No decision status is available
